# Doublecortin-Like Is Implicated in Adult Hippocampal Neurogenesis and in Motivational Aspects to Escape from an Aversive Environment in Male Mice

**DOI:** 10.1523/ENEURO.0324-19.2020

**Published:** 2020-10-14

**Authors:** Dirk-Jan Saaltink, Erik W. van Zwet, Erno Vreugdenhil

**Affiliations:** 1 Department of Cell and Chemical Biology; 2 Department of Biomedical Data Sciences, 2333ZA Leiden University Medical Center, Leiden, The Netherlands

**Keywords:** cognition, DCLK1, doublecortin, hippocampus, neurogenesis, RNA interference

## Abstract

Doublecortin (DCX)-like (DCL) is a microtubule (MT)-associated protein (MAP) that is highly homologous to DCX and is crucially involved in embryonic neurogenesis. Here, we have investigated the *in vivo* role of DCL in adult hippocampal neurogenesis by generating transgenic mice producing inducible shRNA molecules that specifically target DCL but no other splice variants produced by the DCLK gene. DCL knock-down (DCL-KD) resulted in a significant increase in the number of proliferating BrdU+ cells in the subgranular zone (SGZ) 1 d after BrdU administration. However, the number of surviving newborn adult NeuN+/BrdU+ neurons are significantly decreased when inspected four weeks after BrdU administration suggesting a blockade of neuronal differentiation after DCL-KD. In line with this, we observed an increase in the number of proliferating cells, but a significant decrease in postmitotic DCX+ cells that are characterized by long dendrites spanning all dentate gyrus layers. Behavioral analysis showed that DCL-KD strongly extended the escape latency of mice on the circular hole board (CHB) but did not affect other aspects of this behavioral task. Together, our results indicate a function for DCL in adult neurogenesis and in the motivation to escape from an aversive environment. In contrast to DCX, its pivotal role in the maturation of postmitotic neuronal progenitor cells (NPCs) marks DCL as a genuine adult neurogenesis indicator in the hippocampus.

## Visual Abstract

**Figure:**
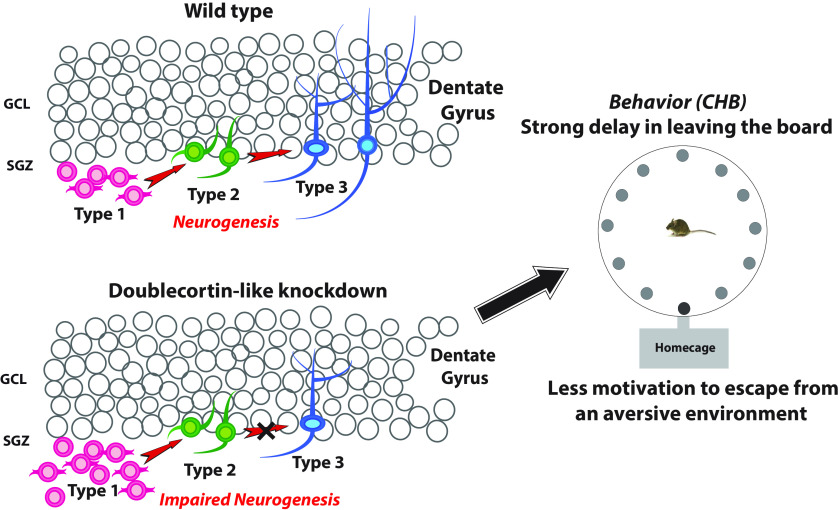

## Significance Statement

Both the doublecortin (DCX) and the DCX-like kinase (DCLK)1 gene are crucial for embryonic neurogenesis. The genomic organization of the DCLK I gene is complex with 20 exons that produces multiple splice variants that are derived from two independent promoters. Whether or not the DCLK1 gene and, if so, which splice variant is involved in adult neurogenesis in the hippocampus is presently unknown. We have investigated specifically the role of one DCLK1 splice-variant, DCL that shares a high level of homology with DCX in both sequence identity and length, in hippocampal neurogenesis. Our data indicate a pivotal role for DCL in adult hippocampus neurogenesis, which is associated with a change in hippocampal memory performance.

## Introduction

The doublecortin (DCX) gene family members are involved in structural plasticity and a rapid adaption of cellular shape (for review, see Reiner et al., 2006). Mutations in the archetypical member of the family, the *doublecortin* (DCX) gene, have been associated with the doublecortex syndrome, which is characterized by aberrant migration of neuroblasts during embryonic development (Gleeson et al., 1998; Francis et al., 1999). Since then, DCX has been extensively used as a marker in the adult central and peripheral nervous system for neurogenesis (Sorrells et al., 2019) and for migrating neuronal progenitor cells (NPCs; Mauffrey et al., 2019). Proteins encoded by this family are microtubule (MT)-associated proteins (MAPs) characterized by a-typical MT binding domains, called DC domains.

Another well-characterized member is the DCX-like kinase (DCLK)1 gene that, like DCX, is necessary for proper neuronal development. Interestingly, like DCX knock-out mice, DCLK1 knock-out mice also lack a clear phenotype (Deuel et al., 2006), but DCLK/DCX double knock-out mice display profound disorganized cortical layering and a disrupted hippocampal structure, suggestive of a compensatory role for the DCLK1 gene in the migration of NPCs during embryogenesis (Deuel et al., 2006; Koizumi et al., 2006). In addition, products of the DCLK1 gene regulate dendritic development *in vitro*, which has been linked with MT-guided transport by DCLK1 interaction with the motor protein kinesin-3 (Liu et al., 2012; Shin et al., 2013; Lipka et al., 2016).

The DCLK gene encodes multiple splice variants encoding proteins containing DC domains and Ser/Thr kinase domains, such as DCLK-long, or Ser/Thr kinase domains only, like DCLK-short (for review, see Dijkmans et al., 2010). In addition, the DCLK gene encodes one splice variant called DCL, that lacks a kinase domain and is highly homologous to DCX over its entire length (Vreugdenhil et al., 2007). During embryonic development, DCL functions as a MT stabilizing protein of mitotic spindles *in vitro* and *in vivo* (Vreugdenhil et al., 2007).

Both DCX and DCL are also expressed in the adult brain. Consistent with a function for DCX in the migration of NPCs, profound DCX expression occurs in well-established neurogenic areas in the adult brain (Brown et al., 2003; Couillard-Despres et al., 2005). DCX+ neuroblasts are well studied in the subgranular zone (SGZ) of the dentate gyrus where ∼20% of the DCX+ cells are proliferating NPCs, while the remaining 80% are postmitotic NPCs and/or neuroblasts (Plümpe et al., 2006; Walker et al., 2007). Surprisingly, DCX seems dispensable for the migration and maturation of (NPCs) and neuroblasts (Merz and Lie, 2013), suggesting that DCL, which is co-expressed with DCX in the SGZ (Saaltink et al., 2012), is sufficient for adult neurogenesis to occur in the dentate gyrus.

Although a role for DCL and the DCLK1 gene in embryonic neurogenesis seems evident (Deuel et al., 2006; Koizumi et al., 2006; Shu et al., 2006; Vreugdenhil et al., 2007), its functional role in adult neurogenesis remains elusive. To address this role, we have generated inducible DCL-shRNA mice to knock-down DCL *in vivo*. As neurogenesis is well-established in the dentate gyrus and DCX and DCL expression is restricted to progenitor cells in the SGZ (Saaltink et al., 2012), we have focused on this neurogenic area of the hippocampus. Furthermore, the cognitive performance after DCL-KD was studied using the CHB paradigm. We report here that inducible knock-down of DCL leads to a dramatic reduction of postmitotic DCX+ cells. In addition, impaired neurogenesis does not affect spatial memory formation. However, DCL-KD leads to a significant increase in the time to escape from the CHB suggesting a subtle role for DCL in context discrimination.

## Materials and Methods

### Animals and animal experimentation

**Figure 1.**
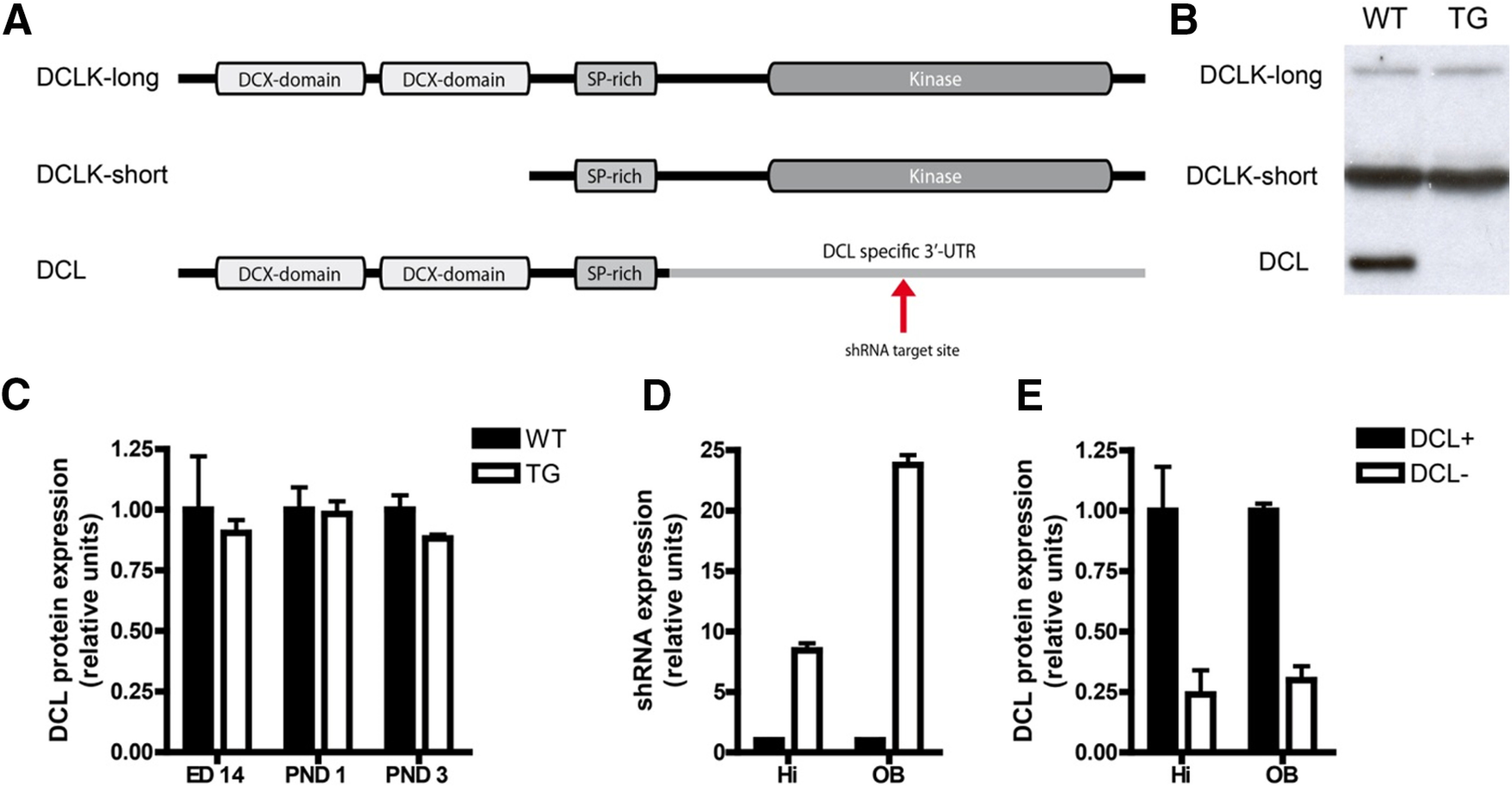
Specific knock-down of DCLK1 splice variant DCL. ***A***, Overview of the three most important DCLK1 splice variants and their functional components. The shRNA target sequence resides in the 3′-UTR of DCL mRNA which is absent in DCLK-long and DCLK-short. ***B***, Western blot analysis reveals splice variant specific knock-down of DCL in dox-induced transgenic (TG) animals compared with dox-induced WT animals. DCLK-long and DCLK-short expression is not affected. ***C***, Although there is some leakage, this leakage does not affect hippocampal DCL expression during embryonic development. There is no significant difference in DCL expression between non-induced WT and TG littermates at embryonic day (ED)14 and postnatal day (PND)1 and PND3. ***D***, After dox induction, in the hippocampal tissue (Hi) an almost 10-fold higher shRNA expression measured compared with non-induced TG littermates (Student’s *t* test, *n* = 4, two-tailed, *p* < 0.0001) In the OB a nearly 25-fold higher shRNA expression is measured (Student’s *t* test, *n* = 4, two-tailed, *p* < 0.0001). ***E***, In both hippocampus (Hi, Student’s *t* test, two-tailed, control *n* = 4, dox *n* = 5, *p* < 0.01) and OB (Student’s *t* test, two-tailed, control *n* = 4, dox *n* = 5, *p* < 0.0001), DCL protein expression is reduced to 25% after dox induction compared with non-induced TG littermates.

Transgenic male mice were obtained from TaconicArtemis GmbH. These mice contain an inducible and reversible shRNA expression system (Seibler et al., 2007), which we called DCL-KD mice. The following hairpin sequences targeting the 3′-untranslated region (UTR) region of the mRNA encoding DCL (Fig. 1*A*) were cloned into the Taconic Artemis system as described previously (Seibler et al., 2007):

5′−TCCC GCTGGTCATCCTGCATCTTGT TTCAAGAGA ACAAGATGCAGGATGACCAGC TTTTTA−3′

3′−CGACCAGTAGGACGTAGAACA AAGTTCTCT TGTTCTACGTCCTACTGGTCG AAAAATGCGC−5'.

Transgenic males were the founders of our heterozygous outbred colony with B6129S6F1 mice. In all our experiments we used males. The shRNA system was induced by doxycycline (dox) via dox containing food pellets (Dox Diet Sterile S3888, 200 mg/kg, BioServ). Animals were put for four weeks on dox diet (*ad libitum*) before they were used for any experiment. As control, we used non-induced TG animals that were fed on identical control diet without dox (S4207, BioServ). As tetracycline-based antibiotics alter mitochondrial function, cell metabolism, cell proliferation, and survival (Ahler et al., 2013; Chatzispyrou et al., 2015; Luger et al., 2018), we used two additional control groups: wild-type (WT) littermates fed with chow and fed with dox containing food pellets.

Tissues are obtained from transgenic DCL-KD mice and WT littermates born in our animal facility. After dox induction animals were decapitated, and brains were quickly removed for dissection of olfactory bulb (OB) and hippocampus. Tissue for qPCR was put into RNA*later* Solution (Applied Biosystems) and kept at 4°C for 1 d and stored at −20°C for later use. Tissue for Western blot analysis was identically dissected, snap-frozen, and stored at −80°C for later use.

All experiments were approved by the local committee of Animal Health and Care and performed in compliance with the European Union recommendations for the care and use of laboratory animals.

### RNA isolation

Total RNA was extracted using TRIzol (Invitrogen) and checked for concentration and purity using a Nanodrop ND-1000 spectrometer (Thermo Scientific). RNA integrity was checked using RNA nano labchips in an Agilent 2100 Bioanalyser (Agilent Technologies, Inc).

To remove genomic DNA, 1 μg RNA of each sample was treated with DNase Amplification Grade (Invitrogen) and diluted with DEPC-MQ to 50 ng/μl RNA. From this purified RNA, cDNA was generated using Bio-Rad iScript cDNA synthesis kit (Bio-Rad).

### shRNA detection

shRNA targeting DCL was measured using a custom designed Taqman miRNA assay on an ABI 7900HT fast real time PCR system (Applied Biosystems). Specific primers were designed to detect anti-DCL shRNA (ACAAGAUGCAGGAUGACCAGC). For mouse tissue, snoRNA-202 was used as reference gene, and the data were analyzed using the ΔΔCt method (Livak and Schmittgen, 2001).

### Western blot analysis

Tissue was solubilized in lysis buffer (1% Tween 20, 1% DOC, 0,1% SDS, 0,15 m NaCl, and 50 mm Tris; pH 7.5) and centrifuged at max speed (14,000 rpm) for 10 min. The protein concentration of the supernatant was measured using the pierce method (Pierce BCA Protein Assay kit, Thermo Scientific). Equal amounts of protein (2 μg cell lysate) were separated by SDS-PAGE (10% acrylamyde) and transferred to immobilon-P PVDF membranes (Millipore).

Blots were incubated in a blocking buffer (tris-buffered saline with 0.2% Tween 20, with 5% low-fat milk powder; TBST) for 60 min and then incubated in fresh blocking buffer with primary antibodies as described (Saaltink et al., 2012) anti-DCL, 1:2000; monoclonal α-tubulin DM1A, 1:10,000; Sigma-Aldrich) for another 60 min. After a 5-min wash (3×) with TBST, horseradish peroxidase-conjugated secondary antibodies were added in TBST. After treatment with 10-ml luminol (200 ml 0.1 m Tris HCl, pH 8, 50 mg sodium luminol, and 60 μl 30% H_2_O_2_), 100 μl Enhancer (11 mg para-hydroxy-coumaric acid in 10 ml DMSO), and 3 μl H_2_O_2_ protein detection, was performed by ECL Western Blotting Analysis System (GE HealthcarePharmacia Biotech).

The developed films were scanned at a high resolution (13,200 dpi) and gray-values were measured using ImageJ. α-Tubulin expression was used to correct for the amount of protein for each sample.

### Histology

#### BrdU treatment

To test whether DCL-KD had an effect on adult neurogenesis, BrdU was used to label proliferating cells. In the first experiment, WT and transgenic animals of six weeks old were put on a dox or control diet (*n* = 6 per group). After four weeks, mice received a single intraperitoneal injection with BrdU (200 mg/kg BrdU dissolved in 0.9% saline, Sigma-Aldrich). After 24 h, the animals were decapitated and prepared for immunohistochemistry as described previously (Saaltink et al., 2012). In a second experiment, animals received a similar diet described above for four weeks. Subsequently, intraperitoneal BrdU (100 mg/kg BrdU dissolved in 0.9% saline, Sigma-Aldrich) was administrated for four consecutive days. The animals were kept on the experimental diet for another four weeks were after the animals were decapitated and prepared for immunohistochemistry as described before.

#### Immunohistochemistry

To measure proliferation, BrdU was visualized with 3,3′-diaminobenzidine (DAB) as previously described (Heine et al., 2004). In short, free-floating sections were incubated in 0.5% H_2_O_2_ to block endogenous peroxidase. Subsequently, the sections were incubated in mouse α-BrdU primary antibody (clone: BMC9318, Roche Diagnostics, 1:1000 overnight) and subsequently in sheep α mouse biotinylated secondary antibody (RPN1001, GE Healthcare, 1:200 for 2 h); both antibodies diluted in 0.1% bovine serum albumin (BSA; sc-2323; Santa Cruz Biotechnology), 0.3% Triton X-100, and 0.1 m phosphate buffer. To amplify the signal, a VectaStain Elite avidin-biotin complex (ABC) kit (Vector Laboratories, Brunschwig Chemie, 1:800 for 2 h) and tyramide (TSA Biotin System, PerkinElmer, 1:750 for 45 min) were used. Thereafter, sections were incubated with DAB (0.5 mg/ml), dissolved in 0.05 m TB with 0.01% H_2_O_2_ for 15 min. Sections were air-dried and counterstained with hematoxylin, dehydrated, and coverslipped with DPX (MerckMillipore).

To analyze cell survival, chicken α-BrdU (ab92837, Abcam, 1:1000) and mouse α-NeuN (MAB3777, Millipore, 1:200) were visualized with fluorescent secondary antibodies (Alexa Fluor 488, goat α-chicken and Alexa Fluor 594 donkey α-mouse, Invitrogen).

To analyze the immature cell population in the dentate gyrus, DCX was visualized with DAB as previously described (Oomen et al., 2007). Briefly, free-floating sections were incubated in 0.5% H_2_O_2_ in 0.05 m TBS (pH 7.6) to block endogenous peroxidase. Before primary antibody incubation, the sections were blocked in 2% low-fat milk powder (Elk, Campina) in TBS for 30 min. Sections were incubated in goat α-DCX (sc-8066; Santa Cruz Biotechnology, 1:800 overnight) and subsequently in biotinylated donkey α-goat (sc-2042; Santa Cruz Biotechnology, 1:500) for 2 h. Both antibodies were diluted in TBS with 0.25% gelatin and 0.1% Triton X-100. To amplify the signal a VectaStain Elite ABC kit and tyramide were used. Incubation of 15 min in DAB (0.5 mg/ml), dissolved in 0.05 m TB with 0.01% H_2_O_2_ finished the staining. Sections were air dried and counterstained with hematoxylin, dehydrated, and coverslipped with DPX.

#### Cell counting

Every tenth section of the collected material (one series out of 10) was stained according the procedures described above. In case of proliferation, all BrdU+ cells in the dentate gyrus were estimated by counting the cells within this series and multiply this with 10. For cell survival, BrdU and NeuN double-positive cells were counted. To analyze the immature population of newborn neurons a distinction based on the dendritic morphology was made between three types of DCX+ cells (Plümpe et al., 2006). We categorized DCX+ cells in proliferative stage (type 1, short of no processes), intermediate stage (type 2, medium processes), and postmitotic stage (type 3, strong dendrites with branches). For all three experiments, the total amount of cells in each section was multiplied by 10.

### Circular hole board (CHB)

#### Apparatus

The CHB paradigm (CHB) was performed as described previously (Dalm et al., 2009). In short, a round Plexiglas plate (diameter: 110 cm) with 12 holes (diameter: 5 cm) was situated 1 m above the floor (Fig. 4*C*). The holes were connected to an s-shaped tube of 15-cm length. Beneath the tube, the home cage was placed such to enable the animal to leave the plate and enter its cage. At 5-cm depth, the holes could be closed by a lid. One week before the experimental procedure, the animals were trained to climb through the tunnel three times.

#### Procedure

At day 1, each mouse started with a free exploration trial (FET) of 300 s. All holes were closed by a lid, and the mouse was allowed to move freely over the board. Seven days after the FET, the animals proceeded with a 4-d training session with two trainings a day (120 s) in which the mice learned to find the exit to their home cage. One day after the training sessions, the animals were once again placed on the board for a FET of 120 s.

#### Behavioral assessments

Video-recorded behavior was automatically analyzed (distance moved, velocity) by Ethovision software (Noldus BV) combined with manually collected data like hole visits, latency to target, and the escape latency. For the latency to target (also mentioned as first visit latency) the time was measured between the start of the trial until placing the nose in the correct hole for the first time. For escape latency the time was measured between the start of the trial until entering the cage. For the automatically analyzed parameters mean distance and mean velocity, the time was taken between the start of the trial until escape from the board or, if this did not happen, the end of the trial (after 120 s).

### Statistics

Results are expressed as mean ± SEM and two-way ANOVA was performed using SPSS statistical software. Behavioral data are tested with a general linear model (GLM) for repeated measurements in SPSS statistical software version 20 (IBM, SPSS Inc.).

## Results

### Generation of DCL-KD mice

To create an inducible DCL-specific knock-down mouse, we designed a shRNA molecule that targets the 3′-UTR of the DCL mRNA that is absent in other splice variants of the DCLK gene (Fig. 1*A*) and has no significant homology with other members of the DCX family. This DCL-specific shRNA was used to generate dox-inducible knock-down mice according standard procedures (Seibler et al., 2007). No obvious phenotypic differences were observed with respect to weight, breeding and behavior in the transgenic DCL-KD mice compared with their littermate WT controls. We checked the expression of DCL-targeting shRNA with or without dox administration by a DCL-specific custom-made qPCR approach. As expected, no shRNA-DCL expression was detected in WT littermate mice (data not shown). Strong hairpin induction was found in both hippocampus and OB of DCL-KD mice (in both cases; Student’s *t* test, *n* = 4, two-tailed, ***p < 0.0001). Compared with transgenic littermates on control diet, a 10-fold (Hi) and 25-fold (OB) higher expression of shRNA was measured in transgenic animals on dox diet (Fig. 1*D*). To investigate specificity of the DCL shRNA, we analyzed the expression of all DCLK1 gene-derived proteins by Western blot analysis. DCL protein levels were reduced to 25% after dox administration in both hippocampus and OB (Fig. 1*E*) while the expression levels of other DCLK1 gene-derived proteins were not affected (Fig. 1*B*). To check for possible fluctuations in DCL expression during neuronal embryogenesis and early postnatal development, a neuronal developmental time-window depending critically on proper expression of DCLK1 gene expression, we inspected DCL expression at embryonic day 14 and postnatal days 1 and 4 by Western blot analysis. We found no significant differences in DCL protein levels in DCL-KD animals compared with their littermate WT controls. Together, we concluded that we generated a reliable mouse model with inducible DCL-specific knock-down.

### DCL knock-down stimulate proliferation but reduces survival of NPCs

**Figure 2.**
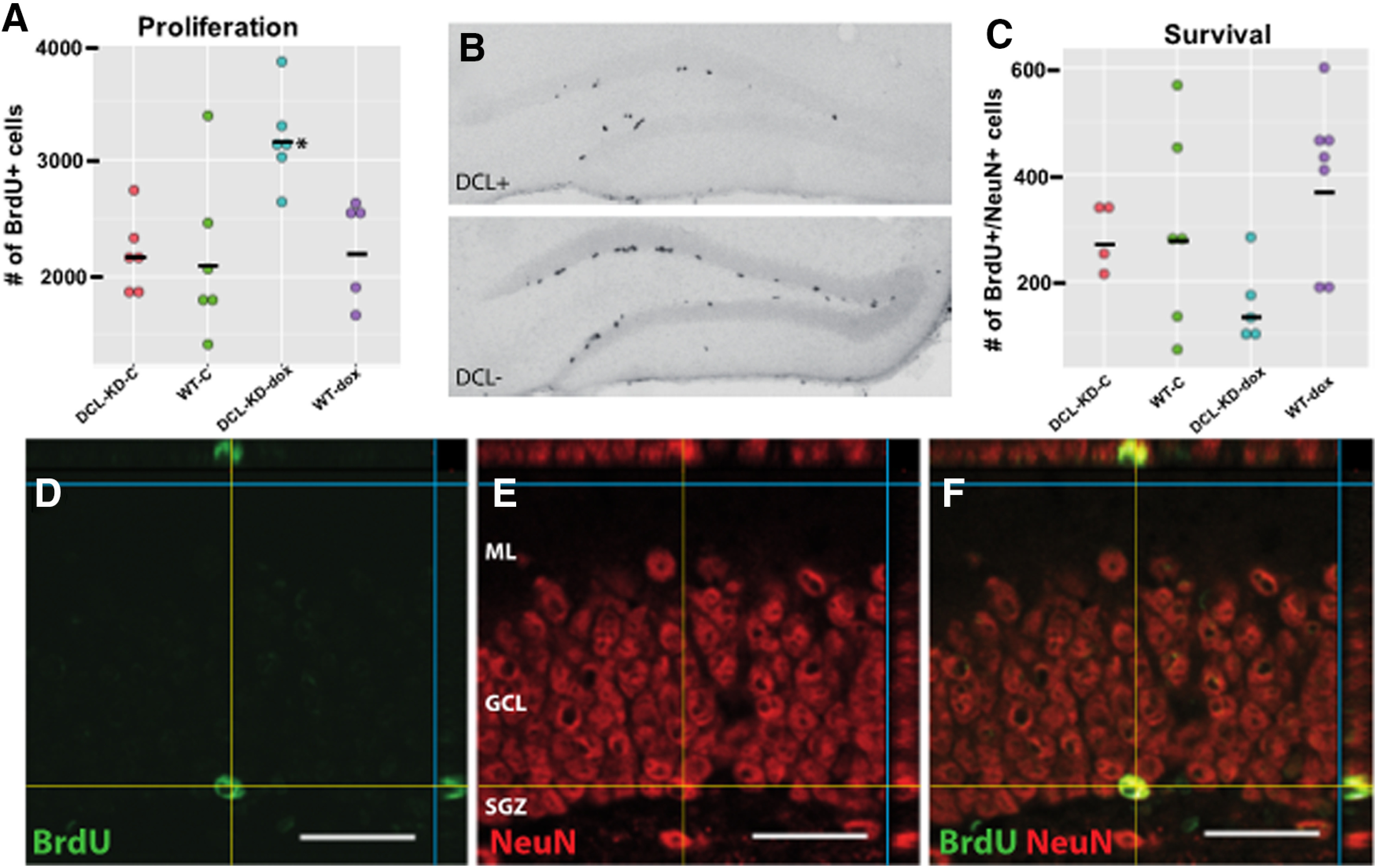
Adult neurogenesis measurement using BrdU labeling. ***A***, Twenty-four hours after a single BrdU injection, a significant (two-way ANOVA, *F*_(3)_ = 6.079, *p* = 0.004) with an significant interaction between genotype and diet (*p* = 0.043). The number of BrdU+ cells is significantly increased compared with WT animals on dox diet and DCL-KD and WT mice on control diets (respectively, *p* = 0.0056, *p* = 0.0022, and *p* = 0.0017; *n* = 6). Effect of dox on DCL-KD mice: 95% CI: 401.7, 1573; *p* = 0.0022). ***B***, Examples of hippocampi derived from animals killed 24 h after BrdU injection. Both sections are stained for BrdU and show mainly BrdU+ cells in the SGZ. Tissue is derived from dox-induced transgenic animals (dox) and non-induced transgenic littermates (control) ***C***, BrdU/NeuN double staining revealed a trend (*F*_(3)_ = 2.77, *p* = 0.057, two-way ANOVA) in double-positive cells in hippocampal dentate gyrus of dox-induced transgenic animals (dox, *n* = 5) compared with non-induced transgenic littermates and both WT control groups (control, *n* = 4). In the dox-fed group, pairwise comparison using *t* tests with pooled SD shows a significant difference between DCL-KD and WT animals (*p* = 0.01). Effect of dox on DCL-KD mice: 95% CI: −323.7, 68.6; *p* = 0.188. ***D–F***, Confocal laser scanning microscopy images showing co localization of BrdU (green in ***D***) and NeuN (red in ***E***). Only cells in the dentate gyrus who are double-positive (yellow in ***F***) were counted. Scale bar in ***D–F***: 25 μm. Significant differences are indicated with an asterisk. Means are indicated with a black bar.

During embryonic development and in cell lines, the DCLK1 gene has been implicated in the formation of mitotic spindles and proliferation of NPCs and in survival of neuroblasts (Vreugdenhil et al., 2007; Verissimo et al., 2010, 2013). Therefore, to investigate the role of the DCL splice-variant in proliferation and survival of adult hippocampal NPCs *in vivo*, we administered the proliferation marker BrdU (Fig. 2*B*,*C*,*E*) to DCL-KD and WT mice and killed these animals after 24 h (proliferation) and after four weeks (survival). For proliferation, a two-way ANOVA revealed a significant effect (*F*_(3)_ = 6.079, *p* = 0.004) with an significant interaction between genotype and diet (*p* = 0.043). Pairwise comparisons using *t* tests with pooled SD showed that the number of BrdU+ cells in DCL-KD animals was significantly increased compared with WT animals on dox diet and DCL-KD and WT animals on control diet (respectively, *p* = 0.0056, *p* = 0.0022, and *p* = 0.0017; Fig. 2*A*). Furthermore, in transgenic DCL-KD mice, the average effect of dox on the outcome BrdU+ NPCs was 987.3 cells [95% confidence interval (CI): 401.7, 1573; *p* = 0.00,224; degrees of freedom (df): 19; Fig. 2*A*]. We measured the survival of newborn NPCs using BrdU in combination with the adult neuron marker NeuN (Fig. 2*D–F*). A two-way ANOVA did not show a significant effect (*F*_(3)_ = 2.77, *p* = 0.07). However, in the dox-fed group, pairwise comparison using *t* tests with pooled SD showed a significant difference between DCL-KD and WT animals (*p* = 0.01; Fig. 2*C*). In transgenic DCL-KD mice, the average effect of dox on the outcome NeuN/BrdU+ neurons was −127.6 cells (95% CI: −323.7, 68.6; *p* = 0.188; df: 18; Fig. 2*C*). Proliferation and cell survival in WT animals were similar as in non-induced transgenic animals. Together, this dataset suggested that proper DCL expression is necessary for NPC survival in the dentate gyrus of the hippocampus.

**Figure 3.**
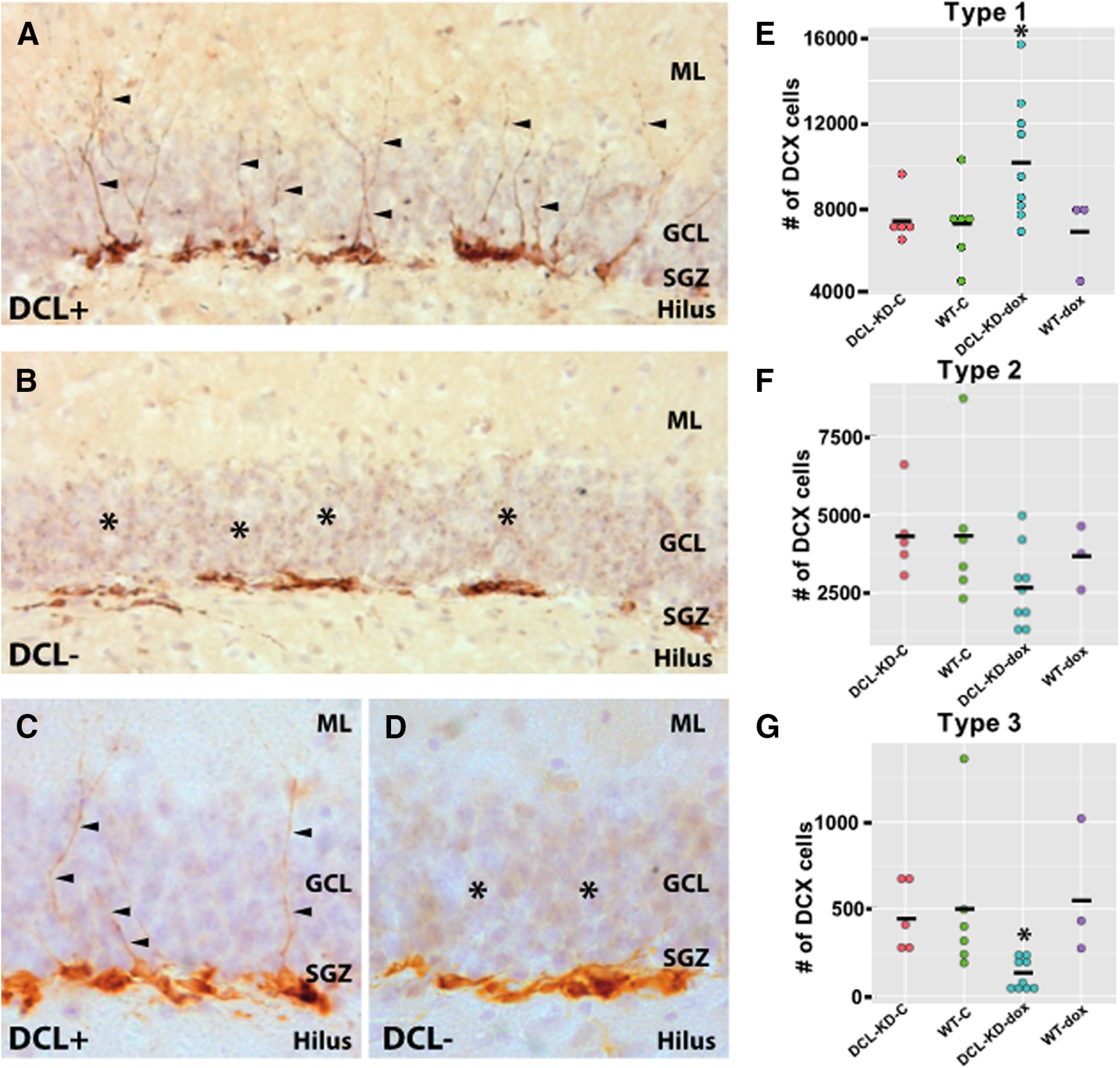
DCX cell morphology. ***A***, DCX-expressing cells in the hippocampal dentate gyrus of a transgenic animal on a control diet showing a normal DCX morphology with cell nuclei close to the SGZ and dendrites toward the molecular layer (ML). ***B***, Hippocampal dentate gyrus of a dox-induced transgenic littermate showing aberrant morphology of DCX+ cells. Hardly any DCX+ cell has dendrites in the granular cell layer (GCL) or ML. ***C***, ***D***, Close-up of DCX-expressing cells in the hippocampal dentate gyrus of a transgenic animal on a control diet (***C***). Several DCX+ cells show dendritic outgrow (arrows) toward the ML which are absent after DCL-KD (***D***). ***E–G***, Number of proliferating type 1, 2, and 3 DCX+ cells in transgenic and WT mice on a control or dox diet. Two-way ANOVA testing shows a significant effect in the type 1 and 3 DCX+ cells (respectively, *F*_(3)_ = 3.377, *p* = 0.04, and *F*_(3)_ = 3.473, *p* = 0.04). Effect of dox on DCL-KD mice: 95% CI type 1 cells: 157.0, 5501.8; *p* = 0.039; 95% CI type 2 cells: −3553.7, 164.5; *p* = 0.072; 95% CI type 3 cells: −665.5, −12.8; *p* = 0.042. Significant differences are indicated with an asterisk. Means are indicated with a black bar. For further details, see main text.

To investigate the role of DCL in neurogenesis in more detail, we labeled NPCs with DCX, a well-established marker for neurogenesis (Brown et al., 2003). The expression of DCX was restricted to three types of proliferating neuronal precursor cells with no or short processes (here called type 1) or medium processes reaching the molecular layer of the dentate gyrus (here called type 2) and postmitotic neuroblasts characterized by elongated dendrites branching into the granule cell layer and molecular layer (here called type 3; categorized after Plümpe et al., 2006; Oomen et al., 2010). Two-way ANOVA testing showed a significant effect in the type 1 and three DCX+ cells (respectively, *F*_(3)_ = 3.377, *p* = 0.04, and *F*_(3)_ = 3.473, *p* = 0.04). Pairwise comparisons using *t* tests with pooled SD revealed that DCL-KD animals had significantly more type 1 DCX+ cells compared with WT animals on dox diet and DCL-KD and WT animals on control diet (respectively, *p* = 0.03, *p* = 0.04, and *p* = 0.02; Fig. 3*E*). The average effect of dox in DCL-KD mice on the outcome type 1 DCX+ NPCs was 2829.4 cells (95% CI: 157.0, 5501.8; *p* = 0.039; df: 19; Fig. 3*A*,*E*). The same pairwise comparison for type 3 cells showed that DCL-KD animals on dox diet have significantly less type 3 cells compared with WT animals on dox diet and DCL-KD and WT animals on control diet (respectively, *p* = 0.03, *p* = 0.04, and *p* = 0.02; Fig. 3*B*,*G*). In transgenic DCL-KD mice, the average effect of dox on the outcome type 3 DCX+ NPCs was −339.2 cells (95% CI: −665.5, −12.8; *p* = 0.042; df: 19; Fig. 3*B*,*G*). A two-way ANOVA did show that there is no effect on type 2 cells between the 4 groups (*F*_(3)_ = 1.824, *p* = 0.18; Fig. 3*F*). In line with this, in transgenic mice, the effect of dox on the outcome type 2 DCX+ NPCs was −1694.6 cells (95% CI: −3553.7, 164.5; *p* = 0. 072; df:19; Fig. 3*D*,*F*). Thus, DCL-KD specifically increased the number of mitotic type 1 cells and reduced the number of postmitotic type 3 cells.

### DCL-KD mice exhibit increased latency to escape from the CHB

Numerous studies indicated that aberrant neurogenesis in the adult hippocampus is associated with disease-associated impaired learning and memory formation (Clelland et al., 2009; Sahay et al., 2011; Fitzsimons et al., 2013; for review, see Samuels and Hen, 2011; Petrik et al., 2012). To investigate possible functional consequences of DCL-KD-induced aberrant neurogenesis, we used the CHB paradigm, a behavioral task aiming to study hippocampal memory performance.

**Figure 4.**
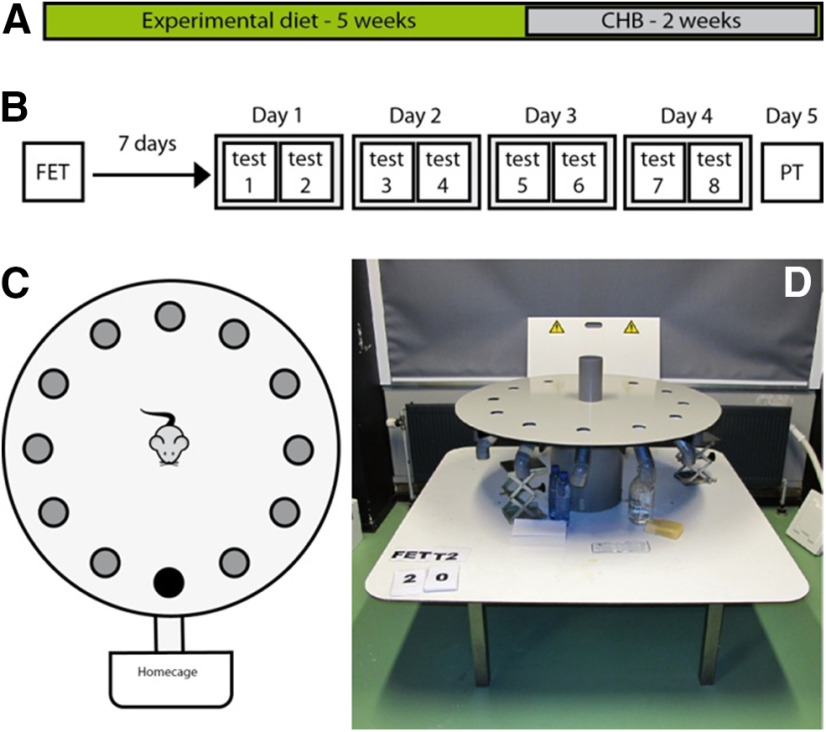
Setup of the CHB experiment. ***A***, Animals were put on a dox diet for at least five weeks before the CHB was started. ***B***, The CHB paradigm started with a FET. Seven days later, the animals followed a training for four consecutive days with two trials a day. At day 5, the animals were exposed to a probe trial in which the escape hole was closed. ***C***, The hole board was equipped with 12 holes. During training, one hole (black), by which animals could reach their home cage, was open. ***D***, Photograph of the CHB setup in the lab.

**Figure 5.**
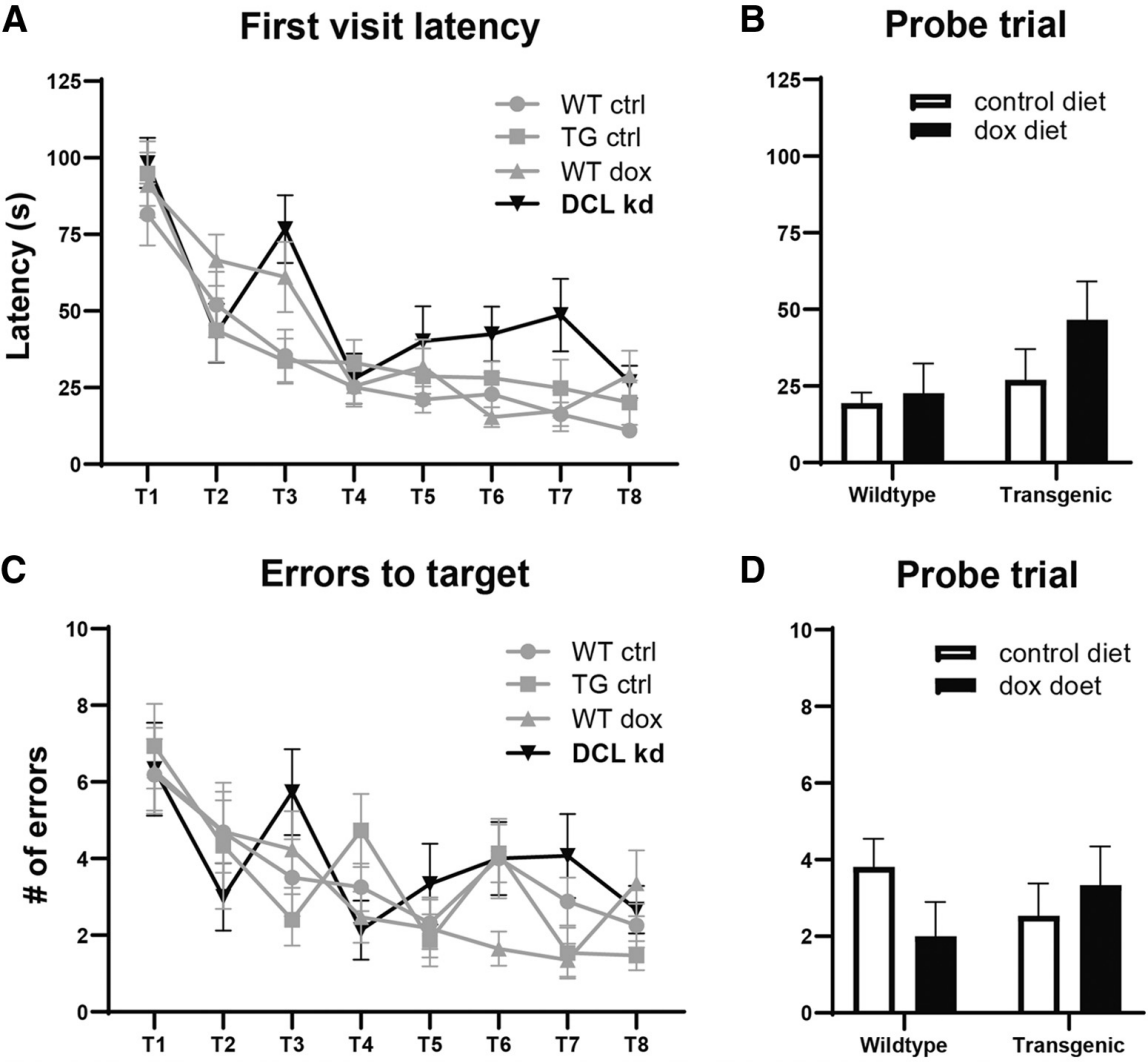
Spatial parameters measured on the CHB. ***A***, First visit latency. All four groups showed a similar decrease over four training days in latency to target (two-way ANOVA for repeated-measures, *F*_(3)_ = 39 521, *p* < 0.001). ***B***, Probe trial. DCL knock-down had no effect on the parameters “latency to target” (two-way ANOVA *F*_(1)_ = 0.744, *p* = 0.392). ***C***, Errors to target. All four groups showed a similar decrease over four training days in errors to target (two-way ANOVA for repeated-measures, *F*_(3)_ = 13.230, *p* < 0.001). ***D***, Probe trial. DCL-KD had no effect on the parameters “errors to target” (two-way ANOVA, *F*_(1)_ = 2222, *p* = 0.141). For further details, see main text.

**Figure 6.**
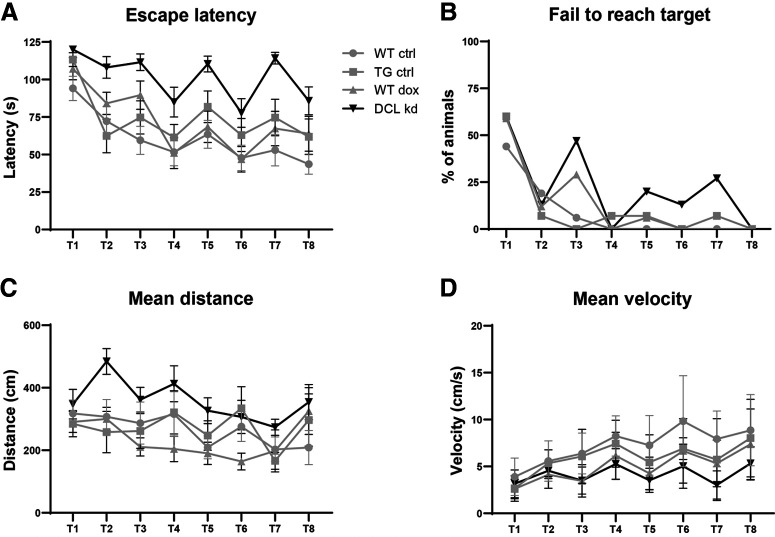
Motivational parameters measured on the CHB. ***A***, DCL-KD animals showed a significant longer escape latency at each first test of the new day (T3, T5, and T7, one-way ANOVA, *F*_(3)_ = 12.574, *p* < 0.005). ***B***, Percent of animals who did not reach the target within 120 s. ***C***, Mean distance moved during each trial. DCL-KD animals move a significant longer distance (two-way ANOVA *F*_(1)_ = 4.366, *p* = 0.041). ***D***, Average velocity during each trial. DCL-KD animals are significant slower compared with DCL+ animals and WT controls (GML, *F*_(1)_ = 15.101, *p* = 0.001).

Four groups (*N* = 16 each), transgenic mice with and without dox and their WT littermate controls were subjected to eight training sessions during four consecutive days followed by a FET with closed exit hole (probe trial: PT; Fig. 4). DCL-KD had no effect on the parameters “latency to” (two-way ANOVA *F*_(1)_ = 0.744, *p* = 0.392; Fig. 5*B*) and “errors to target” (two-way ANOVA, *F*_(1)_ = 2222, *p* = 0.141; Fig. 5*D*) measured during the probe trial. All four groups of mice showed a similar decrease over four training days in latency to target (two-way ANOVA for repeated-measures, *F*_(3)_ = 39 521, *p* < 0.001; Fig. 5*A*) and errors to target (two-way ANOVA for repeated-measures, *F*_(3)_ = 13.230, *p* < 0.001; Fig. 5*C*), suggesting that both groups learned the task equally well. indicating that DCL-KD does not affect spatial learning parameters in the CHB task. However, surprisingly, we observed a highly significant effect on escape latency. All animals showed a learning curve over the four consecutive days (*F*_(3)_ = 11.859, *p* < 0.005) and for each test within 1 d (*F*_(1)_ = 57.136, *p* < 0.005), but overall, there is no interaction effect between gene and diet (two-way ANOVA for repeated-measures, *F*_(3)_ = 1.731, *p* = 0.171; Fig. 6*A*). However, DCL-KD animals had a significant longer escape latency at each first test of a new day (T3, T5, and T7, one-way ANOVA, *F*_(3)_ = 12.574, *p* < 0.005; Fig. 6*A*), whereby DCL-KD animals exhibited a strong delay in leaving the board after finding the exit hole, to their home cage. This finding was supported by the longer moved distance (two-way ANOVA, *F*_(1)_ = 4.366, *p* = 0.041; Fig. 6*C*), and the higher number of animals that failed to reach the target (Fig. 6*B*). This suggested that DCL-KD animals were less motivated to escape from an aversive environment.

## Discussion

Here, we show that DCL is implicated in adult hippocampal neurogenesis. Knock-down of DCL leads to a significant increase in the number of proliferating cells in the SGZ 1 d after BrdU administration. However, the number of newborn adult NeuN+ cells are significantly decreased when studied four weeks after BrdU administration suggesting a suppression of neuronal development after DCL-KD. In line with this, the number of postmitotic DCX+ NPCs are dramatically reduced. As other splice variants of the DCLK1 gene are unaffected and expressed at normal levels, our results demonstrate a role for DCL in the differentiation of newborn neurons that is not compensated for by other DCLK splice variants or other members of the DCX gene family including DCX. Strikingly, DCL-KD strongly reduces the escape latency of mice on the CHB but does not affect other aspects of this behavioral task. Together, our analysis indicates a key role for DCL in cell proliferation, migration, and maturation. DCL is furthermore involved in motivational aspects to escape from an aversive environment.

DCL-KD leads to a significant decrease in the number of postmitotic NeuN+/BrdU+ cells while the number proliferating BrdU+ cells are increased. These data suggest involvement of DCL in cell proliferation and subsequent survival of newborn neurons. Indeed, the DCLK1 gene has been shown to regulate dendritic development (Liu et al., 2012; Shin et al., 2013; Lipka et al., 2016) and the form of mitotic spindles in embryonic NPCs and neuroblasts *in vitro* and *in vivo* (Shu et al., 2006; Vreugdenhil et al., 2007). In *Caenorhabditis elegans*, the orthologue of the DCLK1 gene, zyg-8, regulate asymmetric division of fertilized eggs by controlling the length of mitotic spindles (Gönczy et al., 2001). Also in mammals, a correct positioning of mitotic spindles in radial glia cells has been associated with proper differentiation of the resulting neuronal daughter cells (Lancaster and Knoblich, 2012). Initial neuro-epithelial cell division may occur symmetrical and subsequently, NPCs, i.e., radial glia cells, are believed to divide asymmetrically during embryonic neurogenesis. In analogy with such a proliferation and differentiation scheme, type 1 and type 2 DCX+ cells may represent symmetric dividing progenitor cells in the adult SGZ while type 3 postmitotic DCX+ cells may be the result of an a-symmetric cell division requiring functional DCL. Additionally, The DCLK gene has been shown to be a prosurvival gene in neuroblastoma cells (Kruidering et al., 2001) and is a target for proapoptotic enzymes such as caspases and calpain (Burgess and Reiner, 2001; Kruidering et al., 2001). Moreover, DCLK knock-down by RNA-interference technology leads to the activation of a proapoptotic program in neuroblastoma cells (Verissimo et al., 2010) and to a reduction of NPCs during neocortical development *in vivo* (Vreugdenhil et al., 2007). As the shRNA molecule targets DCL specifically, leaving other DCLK splice variants unaltered, our data indicate a role for DCL in the transition and survival of proliferating to postmitotic DCX+ NPCs.

Knock-down of DCL leads to a phenotypic change of DCX+ cells. This finding suggests that both DCL and DCX are expressed in the same NPCs in the SGZ of the dentate gyrus. In line with DCL/DCX colocalization are the phenotypic analysis of Dcx/Dclk1 double knock-outs mice showing functional redundancy during hippocampal lamination (Tanaka et al., 2006). Also, gene expression profiling of human primary neuroblasts clearly demonstrate coexpression of DCX and DCL (Verissimo et al., 2010). Moreover, our previous immunohistochemical experiments also showed DCX-DCL co-localization in NPCs in the subgranular of the dentate gyrus and in neuroblasts in the rostral migratory stream (Saaltink et al., 2012). Thus, it seems that colocalization of DCX and DCL is required for proper neuronal migration and differentiation. However, at the subcellular level it seems that DCX and DCL are located at different locations with prominent DCX signals that follows projections forming a dendritic blueprint (Fig. 3*A*), while DCL mainly appeared in speckles at specific dendritic hotspots (Saaltink et al., 2012). Also, detailed immunohistochemical analysis during embryonic development shows spatiotemporal differences in expression of DCX and DCL (Boekhoorn et al., 2008). Thus, it seems that DCL and DCX have different subcellular functions in within a cell. However, overexpression or miRNA-mediated DCX knock-down did not alter migration or morphologic maturation of NPCs in the SGZ, suggesting that DCX is dispensable for proper hippocampal neurogenesis (Merz and Lie, 2013). Thus, it seems that DCX and DCL function differently in NPCs with a unique key role for DCL in adequate morphologic maturation.

Previously, we reported a role for DCL in intracellular transport of the glucocorticoid receptor (Fitzsimons et al., 2008), the main mediator of the stress response and a crucial molecule for the migration and maturation of newborn neurons (Fitzsimons et al., 2013). shRNA-mediated glucocorticoid receptor knock-down leads to hyperactive neuronal migration and maturation. Since DCL is directly involved in intracellular GR transport, one might expect similar hyperactive neurogenesis after DCL-KD. However, activated GRs are associated with reduced neurogenesis (Gould et al., 1998) and the increased proliferation after DCL-KD fits into the picture of reduced GR activity. The strongly reduced migration and maturation of NPCs after DCL-KD is opposite to GR knock-down-mediated hyperactive development and suggest that DCL serves more functions beside GR transport. One such function may be mediated by an interaction with members of the kinesin family as DCLK guides kinesin-3 (Lipka et al., 2016) and kinesins have been implicated in the mechanisms underlying asymmetric cell divisions of NPCs (McNeely et al., 2017; Hakanen et al., 2019).

DCL-KD results in aberrant adult neurogenesis but does not affect spatial learning on the CHB. This finding is somewhat unexpected as several studies reported association of reduced neurogenesis and impaired spatial and contextual learning in several behavioral tasks such as contextual fear conditioning (Seo et al., 2015) and, similar as the CHB, the Barnes maze (Imayoshi et al., 2008). However, these findings were not reproduced by numerous other investigators (Shors et al., 2002; Meshi et al., 2006; Zhang et al., 2008; Martinez-Canabal et al., 2013). For example, even complete ablation of neurogenesis in cyclin D2 knock-out mice leads to normal spatial learning and contextual memory formation (Jaholkowski et al., 2009; Jedynak et al., 2012; Urbach et al., 2013). Moreover, addition of new neurons is not necessary for hippocampus-dependent learning (Frankland, 2013) but may be involved in forgetting, although this is dependent on the memory task used and its timing in relation to neurogenesis (Gao et al., 2018). Recent studies suggest a role for adult neurogenesis in a more subtle cognitive hippocampal function, i.e., pattern separation (Clelland et al., 2009; Sahay et al., 2011; França et al., 2017). Thus, the CHB paradigm may be too robust to find possible cognitive hippocampus-mediated impairments after DCL-KD. Alternatively, DCL-KD leads to ∼75% reduction of adult-born postmitotic neurons (Fig. 3*G*), which may be insufficient to detect neurogenesis-related behavioral differences.

Surprisingly, DCL-KD leads to a highly significant increase in the latency to leave the CHB. Possibly, motivation to leave the CHB, might be fear-regulated by the aversive environment created by the board and as such, comparable with context fear conditioning which may be partly regulated by adult neurogenesis (Drew et al., 2010; Denny et al., 2012). Also, this increase in latency is associated with more motor activity with longer moved distances after DCL-KD, a phenomenon that is also linked to a lesioned hippocampus (Deacon et al., 2002). Alternatively, although DCL has a highly restrictive expression pattern in the hippocampus (Saaltink et al., 2012), we cannot exclude the possibility that other brain areas are involved. In particular, DCL is also highly expressed in the OB. Ablation of newly born neurons does not affect olfactory detection levels, however, it might affect downstream processing of odour information (Gheusi et al., 2000; Imayoshi et al., 2008) and as such DCL knock-down might impair olfactory discrimination. Therefore, impaired olfaction might result in impaired recognition of the home cage, which might explain the increased latency to leave the board. However, olfaction is an equally important parameter to learn spatial memory tasks adequately (van Rijzingen et al., 1995; Machado et al., 2012). Moreover, we did not observe any differences, as in the hippocampus, in the form and number of DCX+ cells in the OB (Saaltink and Vreugdenhil, unpublished data) while DCL is also expressed in other brain areas characterized by a high level of neuronal plasticity (Saaltink et al., 2012). Therefore, we favor the hypothesis that the increase in latency is because of impaired structural alterations in the dentate gyrus.

We have successfully generated a transgenic animal model to study the role of a specific splice-variant of the DCLK gene, i.e., DCL, without affecting the expression of the other splice variants DCLK-long and DCLK-short. Using this model, we found that DCL is involved in the transition of proliferating NPCs into postmitotic neuroblasts. Moreover, behavioral studies show that DCL may be involved in motivational aspects to escape from aversive environments. Our model seems a valuable *in vivo* tool to study these areas and the role of DCL therein, in a multidisciplinary fashion.

## Synthesis

Reviewing Editor: Rae Silver, Columbia University Decisions are customarily a result of the Reviewing Editor and the peer reviewers coming together and discussing their recommendations until a consensus is reached. When revisions are invited, a fact-based synthesis statement explaining their decision and outlining what is needed to prepare a revision will be listed below. The following reviewer(s) agreed to reveal their identity: Russell Romeo.

Overview: The study involves a novel model that can be used to study the function of doublecortin-like protein (DCL) in adulthood, or during specified developmental time periods. More specifically, the manuscript reports on the effects of knocking down (via inducible shRNA) doublecortin-like (DCL) expression on hippocampal cellular proliferation, neurogenesis, and performance on the circular hole board (CHB) test in male mice. The authors report that DCL knockdown leads to enhanced cellular proliferation, but reduced survival of newly born neurons in the dentate gyrus. Moreover, despite these cellular changes, spatial learning (as indexed by CHB performance) was unaffected, but motivation to escape an aversive environment was compromised. This manipulation in adult mice is novel and provides a helpful model to understand the role of DCL in neurogenesis related processes in adulthood. The authors clearly document the efficacy of the Knockdown manipulation on DCL expression, validating the model for use in this and future studies. Overall, the paper is well written and the data are both interesting and novel.

Major comments:

1. While the effect of DCL knockdown on cell proliferation and survival in the Dentate gyrus are clearly described, the results are only partially presented. According to the methods, in both assays (proliferation and survival), both WT and TG mice are treated with either a Dox or no dox diet. However only the data from TG mice is presented. A statement is provided that:

"Proliferation and cell survival in Wildtype animals were similar as in non-induced transgenic animals”.

This data should be shown because it is known that Tetracycline based antibiotics alter mitochondrial function, cell metabolism, cell proliferation and survivial (see references pasted below). Therefore, is it critical that the experimental design includes a control for the effect of the Dox diet. The most appropriate analysis would be ANOVA with 2 factors (genotype and diet). This is also the case for the analysis of the 3 types of neuroprogenitor cells quantified in DCL KD mice (figure 3).

References

Ahler E, Sullivan WJ, Cass A, Braas D, York AG, Bensinger SJ, Graeber TG, Christofk HR. Doxycycline alters metabolism and proliferation of human cell lines. PLoS One. 2013 May 31;8(5):e64561. doi: 10.1371/journal.pone.0064561.Print 2013. PubMed PMID: 23741339; PubMed Central PMCID: PMC3669316.

Chatzispyrou IA, Held NM, Mouchiroud L, Auwerx J, Houtkooper RH. Tetracycline antibiotics impair mitochondrial function and its experimental use confounds research. Cancer Res. 2015 Nov 1;75(21):4446-9. doi: 10.1158/0008-5472.CAN-15-1626. Epub 2015 Oct 16. Review. PubMed PMID: 26475870; PubMed Central PMCID: PMC4631686.

Moullan N, Mouchiroud L, Wang X, Ryu D, Williams EG, Mottis A, Jovaisaite V, Frochaux MV, Quiros PM, Deplancke B, Houtkooper RH, Auwerx J. Tetracyclines Disturb Mitochondrial Function across Eukaryotic Models: A Call for Caution in Biomedical Research. Cell Rep. 2015 Mar 17;10(10):1681-1691. doi: 10.1016/j.celrep.2015.02.034. Epub 2015 Mar 12. PubMed PMID: 25772356; PubMed Central PMCID: PMC4565776.

Luger AL, Sauer B, Lorenz NI, Engel AL, Braun Y, Voss M, Harter PN, Steinbach JP, Ronellenfitsch MW. Doxycycline Impairs Mitochondrial Function and Protects Human Glioma Cells from Hypoxia-Induced Cell Death: Implications of Using Tet-Inducible Systems. Int J Mol Sci. 2018 May 17;19(5). pii: E1504. doi: 10.3390/ijms19051504. PubMed PMID: 29772845; PubMed Central PMCID: PMC5983704.

2. 2-factor ANOVA should be used to determine if there is an interaction between genotype and diet. ( i.e. to control for the effect of DOX), as explained in the review below

3. The Results of the spatial memory test show an effect of DCL-KD, or dox. Here the authors state that:

"As both wildtype groups were indistinguishable from the transgenic mice without dox, for reasons of clarity we only present here the with (DCL-KD) and without dox (DCL+) transgenic groups (data not shown). Again, because of the known effects of Dox, it is important to show the results of all 4 groups, or, provide a summary of the statistical analysis that justifies presenting only the data from the two transgenic groups.

4. The interpretation that DCL-KD reduces motivation to escape an aversive environment is certainly possible but not particularly convincing based on the data presented. First, the parameters analyzed in in the circular hole board task are not well described and are not the same parameters analyzed in the paper cited for the CHB method. For example, was “latency to target” the time taken from start until placing the nose in the correct hole? While latency to escape is the interval between start and entering the cage? In which case, does “mean distance” and “mean velocity” include distance and time travelled after placing the nose in the correct hole, but continuing to explore the board before entering the tunnel?

Also, what is the latency to begin searching for the escape hole (movement initiation)? this would presumably also reflect a reduction in motivation to escape. What is aversive about the CHB in this study? There is no mention of noise or bright lights in the method section and in the paper cited for the method (Dalm et al., 2009), no such aversive stimuli were used.

In the discussion, the authors consider a plausible explanation why memory (measured by latency and errors to choice) in the CHB is unaffected: the CHB is relatively easy. The authors also consider why escape distances and latencies might be altered: changes in hippocampal based fear-related processes or changes in olfaction). These are interesting points of discussion.

Minor points:

The presentation of the data in figs 5 and 6 is somewhat confusing because in A and C, DCL+ is black and DCL- is grey but in B and D, DCL- is black and DCL+ is clear. The key should be consistent within fig 5, and also with fig 6.

List of typos:

De archetypal = the archetypal

4 Consequential days = 4 consecutive days

Lith = stone? Lid? Door?

a-symmetric = asymmetric

Title: should indicate that these results pertain to male mice.

Abstract: indicates that “DCL-KD strongly reduced the escape latency...”, but the data appear to show the opposite. Please clarify.

Methods: should it be: closed by a lid?

Results: is the “1.51 more” a percent? Similarly “1.2 more” and “0.61 less” are these percent as well?

Results: the authors indicate that there are two types of proliferating neuronal precursor cells, but then go on to classify them into type 1, type 2, and type 3 cells. Please clarify.

Once circular hole board is defined as CHB, the authors should use CHB consistently throughout the paper.

Given the reduced survival of newly born neurons, do these mice with DCL knockdown show any reduced cell density in the dentate gyrus or reduced dentate gyrus volume/thickness?

## Author Response

Dear Dr. Silver,

Please find enclosed our revised manuscript entitled ‘"Doublecortin-like is implicated in adult hippocampal neurogenesis and in motivational aspects to escape from an aversive environment in male mice” (eNeuro eN-NWR-0324-19). We like to thank both reviewers for their constructive criticism that considerably improved the content of our manuscript.

We have addressed all comments of both reviewers and indicated our textual adaptions in red. We have adjusted our manuscript on the following points:

Major comment:

1. While the effect of DCL knockdown on cell proliferation and survival in the Dentate gyrus are clearly described, the results are only partially presented. According to the methods, in both assays (proliferation and survival), both WT and TG mice are treated with either a Dox or no dox diet. However only the data from TG mice is presented. A statement is provided that: “Proliferation and cell survival in Wildtype animals were similar as in non-induced transgenic animals”. This data should be shown because it is known that Tetracycline based antibiotics alter mitochondrial function, cell metabolism, cell proliferation and survival (see references pasted below). Therefore, is it critical that the experimental design includes a control for the effect of the Dox diet. The most appropriate analysis would be ANOVA with 2 factors (genotype and diet). This is also the case for the analysis of the 3 types of neuroprogenitor cells quantified in DCL KD mice (figure 3).

Our reply:

As requested, we have included data from the two additional groups (WT mice fed with a dox diet and WT mice fed with a doxycycline diet). The data are incorporated in Figure 2 and 3 and are described in the results section. We have also incorporated the references, kindly provided by the reviewers, in the material and method section. For the statistical testing of our results we have consulted Dr Erik van Zwet from our department of Biomedical Data Science and =as requested- applied 2-way ANOVA. We have indicated this statistical testing in material and methods, the results section and in the legends of the different figures.

Major comment:

2. 2-factor ANOVA should be used to determine if there is an interaction between genotype and diet. (i.e. to control for the effect of DOX), as explained in the review below

Our reply:

See also our reply above. We have re-analyzed our data of the four groups of mice with 2-factor ANOVA.

3. The Results of the spatial memory test show an effect of DCL-KD, or dox. Here the authors state that:

"As both wildtype groups were indistinguishable from the transgenic mice without dox, for reasons of clarity we only present here the with (DCL-KD) and without dox (DCL+) transgenic groups (data not shown). Again, because of the known effects of Dox, it is important to show the results of all 4 groups, or, provide a summary of the statistical analysis that justifies presenting only the data from the two transgenic groups.

Our reply:

As requested, we now show the results of all 4 groups in Figure 5 and 6 and have adjusted the corresponding text.

Major comment:

4a. The interpretation that DCL-KD reduces motivation to escape an aversive environment is certainly possible but not particularly convincing based on the data presented. First, the parameters analyzed in in the circular hole board task are not well described and are not the same parameters analyzed in the paper cited for the CHB method. For example, was “latency to target” the time taken from start until placing the nose in the correct hole? While latency to escape is the interval between start and entering the cage? In which case, does “mean distance” and “mean velocity” include distance and time travelled after placing the nose in the correct hole, but continuing to explore the board before entering the tunnel?

Our reply:

In the method section, under ‘Behavioral assessments’, we have extended the description of the analyzed parameters. Indeed, we have defined ‘latency to target’ as the time between the start of the trial and the placement of the nose in the correct hole. Mean distance and mean velocity are defined as the time between start of the trial until escape from the board.

Major comment:

4b. Also, what is the latency to begin searching for the escape hole (movement initiation)? this would presumably also reflect a reduction in motivation to escape. What is aversive about the CHB in this study? There is no mention of noise or bright lights in the method section and in the paper cited for the method (Dalm et al., 2009), no such aversive stimuli were used.

In the discussion, the authors consider a plausible explanation why memory (measured by latency and errors to choice) in the CHB is unaffected: the CHB is relatively easy. The authors also consider why escape distances and latencies might be altered: changes in hippocampal based fear-related processes or changes in olfaction). These are interesting points of discussion.

Our reply:

As mentioned above, we measured the time between start of the trial and placing the nose in the correct hole. We did not discriminate the latency to begin searching for the escape hole and therefore, unfortunately, we cannot provide these data. However, we like to point out that we did not observe differences in the four groups in the free exploration time and in the first day of testing. Also, we performed our CHB experiments based on the paper by Sergiu Dalm (see p102. Dalm et al, 2009. in our manuscript), who considered the CHB test as anxiety-related: ‘Spending more time in the central, most unprotected area is generally accepted as reduced anxiety-like behaviour (Archer J. 1973. Anim. Behav. 21: 205-235; Choleris et al, 2001. Neurosci. Biobehav. Rev. 25: 235-260). On the CHB this will increase the latency to the exit hole’.

Minor points:

The presentation of the data in figs 5 and 6 is somewhat confusing because in A and C, DCL+ is black and DCL- is grey but in B and D, DCL- is black and DCL+ is clear. The key should be consistent within fig 5, and also with fig 6.

Our reply:

We have adjusted Figure 5 and 6 according the comments of the reviewer.

List of typos:

De archetypal = the archetypal

4 Consequential days = 4 consecutive days

Lith = stone? Lid? Door?

a-symmetric = asymmetric

Our reply:

All 4 typo’s are corrected. ‘Lith’ is replaced by ‘lid’

Title: should indicate that these results pertain to male mice.

Our reply:

As requested, we have added ‘in male mice’ in the title.

Abstract: indicates that “DCL-KD strongly reduced the escape latency...”, but the data appear to show the opposite. Please clarify.

Our reply:

We like to thank the reviewer for this important correction. We have replaced ‘reduced’ by ‘extended’

Methods: should it be: closed by a lid?

Our reply:

Indeed, we have added ‘by a lid’ in the Method section.

Results: is the “1.51 more” a percent? Similarly “1.2 more” and “0.61 less” are these percent as well?

Our reply:

We have analyzed our data with 2-way ANOVA, and these figures are no longer relevant for the paper and have been removed.

Results: the authors indicate that there are two types of proliferating neuronal precursor cells, but then go on to classify them into type 1, type 2, and type 3 cells. Please clarify.

Our reply:

‘Two types of proliferating neuronal precursor cells’ is a mistake from our site. This should be three types of NPC’s and has now been corrected in the revised manuscript.

Once circular hole board is defined as CHB, the authors should use CHB consistently throughout the paper.

Our reply:

We have replaced ‘circular hole board’ by ‘CHB’ throughout the manuscript.

Given the reduced survival of newly born neurons, do these mice with DCL knockdown show any reduced cell density in the dentate gyrus or reduced dentate gyrus volume/thickness?

Our reply:

This is indeed a very interesting point. However, to investigate DG volume/thickness requires extensive stereological studies, which we believe is outside the scope of our study.
